# Midwife-led birthing centres in Bangladesh, Pakistan and Uganda: an economic evaluation of case study sites

**DOI:** 10.1136/bmjgh-2023-013643

**Published:** 2024-03-28

**Authors:** Emily J Callander, Vanessa Scarf, Andrea Nove, Caroline Homer, Alayna Carrandi, Abu Sayeed Abdullah, Sheila Clow, Abdul Halim, Scovia Nalugo Mbalinda, Rose Chalo Nabirye, AKM Fazlur Rahman, Saad Ibrahim Rasheed, Arslan Munir Turk, Oliva Bazirete, Sabera Turkmani, Mandy Forrester, Shree Mandke, Sally Pairman, Martin Boyce

**Affiliations:** 1 Faculty of Health, University of Technology Sydney, Sydney, New South Wales, Australia; 2 Novametrics Ltd, Duffield, UK; 3 Burnet Institute, Melbourne, Victoria, Australia; 4 Monash University School of Public Health and Preventive Medicine, Melbourne, Victoria, Australia; 5 Centre for Injury Prevention and Research, Dhaka, Bangladesh; 6 University of Cape Town, Cape Town, South Africa; 7 Makerere University, Kampala, Uganda; 8 Busitema University, Tororo, Uganda; 9 Research and Development Solutions, Islamabad, Pakistan; 10 University of Rwanda, Kigali, Rwanda; 11 International Confederation Of Midwives, The Hague, The Netherlands

**Keywords:** Maternal health, Health economics

## Abstract

**Introduction:**

Achieving the Sustainable Development Goals to reduce maternal and neonatal mortality rates will require the expansion and strengthening of quality maternal health services. Midwife-led birth centres (MLBCs) are an alternative to hospital-based care for low-risk pregnancies where the lead professional at the time of birth is a trained midwife. These have been used in many countries to improve birth outcomes.

**Methods:**

The cost analysis used primary data collection from four MLBCs in Bangladesh, Pakistan and Uganda (n=12 MLBC sites). Modelled cost-effectiveness analysis was conducted to compare the incremental cost-effectiveness ratio (ICER), measured as incremental cost per disability-adjusted life-year (DALY) averted, of MLBCs to standard care in each country. Results were presented in 2022 US dollars.

**Results:**

Cost per birth in MLBCs varied greatly within and between countries, from US$21 per birth at site 3, Bangladesh to US$2374 at site 2, Uganda. Midwife salary and facility operation costs were the primary drivers of costs in most MLBCs. Six of the 12 MLBCs produced better health outcomes at a lower cost (dominated) compared with standard care; and three produced better health outcomes at a higher cost compared with standard care, with ICERs ranging from US$571/DALY averted to US$55 942/DALY averted.

**Conclusion:**

MLBCs appear to be able to produce better health outcomes at lower cost or be highly cost-effective compared with standard care. Costs do vary across sites and settings, and so further exploration of costs and cost-effectiveness as a part of implementation and establishment activities should be a priority.

**WHAT IS ALREADY KNOWN ON THIS TOPIC**

- Midwife-led birth centres (MLBCs) have promising clinical evidence to support their implementation in low-income and middle-income countries, but there is an absence of evidence for costs and cost-effectiveness of implementing MLBCs relative to standard care.

**WHAT THIS STUDY ADDS**

- This economic evaluation is the first study to quantify the real-word operation costs of MLBCs outside of high-income country settings. Our findings from Bangladesh, Pakistan and Uganda showed MLBCs can be cost-saving or cost-effective relative to standard care, and thus appear to be broadly consistent with results from other high-income country settings.

**HOW THIS STUDY MIGHT AFFECT RESEARCH, PRACTICE OR POLICY**

- Our methodology, including a codesigned data collection tool with country researchers, highlighted the importance of close collaboration with local health service teams to identify the context of expenditure. The implementation of MLBCs in low-income and middle-income countries could be cost saving and cost-effective at small or larger scales, once contextual factors are considered.

## Introduction

The United Nations has set targets within the Sustainable Development Goals (SDGs) to reduce maternal and neonatal mortality.^1^ Also featured in the SDGs is universal access to healthcare—ensuring all people, regardless of location, have access to affordable and appropriate healthcare.^1^ Achieving these dual goals is a challenge for all countries, particularly low-income and middle-income countries (LMICs), where maternal and neonatal mortality is highest,^2 3^ as this will generally require improving service access and quality, alongside expanding services.

Increasing and promoting facility-based birth has been the main strategy for reducing maternal and neonatal mortality in many LMICs.^4^ However, increased rates of births in a facility do not directly translate to reduced mortality if the facilities provide poor-quality care.^5^ Regional and global disparities in maternity care across wealth quintiles and geographical locations,^5^ alongside service challenges regarding funding and resources (including staffing and training),^6^ pose significant hurdles to upscaling access to safe, high-quality maternity care.

High-income countries have increasingly taken a medicalised approach to maternity care.^7 8^ While this approach sees low mortality rates,^8^ there is a concern that the pendulum has swung too far. High rates of medical intervention during childbirth, such as caesarean birth and labour inductions, have led to short-term and long-term harms^9–12^ and high and rapidly increasing costs per birth,^10 12 13^ which may be becoming unaffordable even in high-income countries.^13^ While many lessons can be learnt from models of care in high-income countries,^8^ these may not represent the most effective and efficient path forward to achieving the SDGs in LMICs.

Midwife-led birth centres (MLBCs), where the lead healthcare professional at the time of birth is a midwife, are often seen as an alternative to hospital-based care for low-risk pregnancies and have been used in many countries.^14^ This model of care been associated with increased rates of maternity service utilisation and reported satisfaction among women, strengthened networks of care and reduced rates of unnecessary interventions during childbirth.^14^ As such, MLBCs may offer an appropriate option for providing maternity care in LMICs for women with uncomplicated pregnancies. There is, however, an absence of evidence about the costs associated with the establishment and operation of MLBCs and estimates around their cost-effectiveness relative to standard care in LMICs.

The objective of this study was to identify the costs of operating MLBCs in real-world LMIC settings, and to estimate their cost-effectiveness relative to standard care. We used a case study approach, with 4 MLBC sites in Bangladesh, Pakistan and Uganda (12 sites in total) to collect data on costs and outcomes of MLBCs and conduct a modelled cost-effectiveness analysis. The purpose of the study was to inform decision-making about the expansion of this model of care. The decision-making questions were as follows: (1) what would it cost to operate additional MLBCs in LMICs and (2) what would be the cost-effectiveness of additional MLBCs in LMICs?

## Methods

### Study setting and location

Bangladesh, Pakistan and Uganda were selected to participate in this study, based on the findings of a global literature review and survey^15^ and consultation with the project’s advisory group. The advisory group consisted of experts in MLBCs from high-income, middle-income and low-income contexts and representatives of the International Confederation of Midwives, WHO, United Nations Population Fund, Bill & Melinda Gates Foundation and World Bank. The main inclusion criteria were as follows: (a) the country was classed by the World Bank in 2022 as low-income, lower-middle-income or upper-middle-income; (b) there was evidence from the literature and the survey that the country had at least four MLBCs that were either in the public sector or well integrated within the national health system; (c) good research capacity within the country and (d) data were expected to be available for this economic analysis. Each country that met the inclusion criteria was invited to participate through the national Ministry of Health and through the International Confederation of Midwives member association(s). National research teams were recruited by the International Confederation of Midwives, and these teams identified four MLBC sites per country for inclusion. Site selection was based on a combination of representativeness and feasibility and informed by a desk review of the literature and consultation with the national Ministry of Health, the International Confederation of Midwives member association, the national research team, the site manager(s) and other relevant stakeholders.

### Study population

For the purposes of this study, we adopted the following definition of an MLBC: a dedicated space offering childbirth care, in which midwives take primary clinical responsibility for birthing care. Antenatal and postpartum care may also have been provided, but this was not essential for classification as an MLBC. Most of the 12 MLBC sites (n=10 sites), including all the Ugandan MLBCs, were freestanding, that is, on a site separate from a health facility to which the MLBC could refer women if needed. The remaining MLBCs (n=2 sites) were on the same site as a referral facility (online supplemental appendix 1). Most MLBCs (n=8 sites) were in the private sector (including for-profit and not-for-profit), two were public–private partnerships (ie, public-sector facilities supported by non-governmental organisations) and two were in the public sector.

10.1136/bmjgh-2023-013643.supp1 Supplementary data

### Comparator

The comparison was current ‘standard care’ in each country. This could have included a combination of hospital-based birth and home birth. As the decision-making question was concerned with expansion of MLBCs within the local setting, this heterogeneity in comparison was considered appropriate.

### Study design

We conducted a cost analysis of MLBCs using primary data collection from routine data captured by the MLBCs in each of the four country sites within each of the three countries. Data were collected between October and December 2022. The data collection tool covered costs of operating the MLBC and outcomes of women and was codesigned with study teams from each country to ensure data availability. Data items related to costs of facility operation included utilities, staff salaries, staff training and equipment purchase and hire (online supplemental appendix 2). The included costs represent the annual costs of operating an MLBC. Facility purchase costs were considered sunk costs and not included. Data related to health outcomes included transfer to other facilities, caesarean birth at other facilities, morbidity (eg, incidence of haemorrhage, third or fourth degree tears or other serious morbidities), maternal mortality, stillbirth, neonatal mortality and any costs paid by the women or their families.

A modelled cost-effectiveness analysis was then conducted, comparing MLBCs with standard care. This took the form of a decision analysis tree with 1000 hypothetical women (online supplemental appendix 3). Women entered the model immediately prior to birth. In the MLBC arm, they then were either transferred or gave birth at the facility. All women who were transferred then had either a vaginal birth or caesarean birth and then had either no morbidities or morbidities. All women who gave birth in an MLBC had a vaginal birth and then had either no morbidities or morbidities. Data for the MLBC arm were collected from primary data from study sites. For current standard care within each country, rates of caesarean birth, stillbirth and neonatal death were obtained from the UNICEF Data Warehouse.^16^ Rates of maternal mortality were obtained from WHO modelled estimates,^2^ and maternal morbidity rates were obtained from the literature (online supplemental appendix 4).^17 18^

Per-woman costs for the operation of the MLBC were added to women in the MLBC arm, based on reported costs from the primary data collection. A cost for transfer was obtained from the primary data collection and applied to those who were transferred from the MLBC to another facility after onset of labour. For women in both arms who had a caesarean birth in a non-MLBC institution (MLBCs do not offer caesarean sections, because this procedure is not within the scope of practice of a midwife), costs per caesarean birth were sought for each country from the literature. Costs were separated into costs paid by the health service and out of pocket costs incurred by women (online supplemental appendix 5).

Disability-adjusted life-years (DALYs) were allocated based on morbidity rates for the women and mortality rates for women and newborns. Categories were no maternal morbidity, maternal morbidity, maternal mortality and stillbirth or neonatal death (online supplemental appendix 6). Disability weights for maternal morbidity were obtained from the average of the following conditions, identified from the Global Burden of Disease Study^19^ and were calculated based on the average weight for maternal haemorrhage, pregnancy-related sepsis, hypertensive disorders, obstructed labour, rectal fistula and vesicovaginal fistula.

### Patient and public involvement in research

The cost data collection tool was codesigned by the research team and the national researchers. The national researchers engaged with each of the MLBC sites to identify typical annual expenditures. After initial data collection, a series of meetings were held between the analysis team and the national research teams to validate the data provided.

### Time horizon and discount rate

We adopted a health funder perspective. The time horizon for the cost and cost-effectiveness analysis was 1 year, and as such no discounting was required. The short 1-year time horizon is considered conservative and underestimates the value of health outcomes produced over a lifetime; however, due to the absence of primary data collection for health outcomes this was considered necessary to avoid introducing additional uncertainty.

### Currency, price date and conversion

All costs are presented in 2022 US dollars. Costs were inflated to 2022 dollars based on published inflation rates and converted from original currency to US dollars based on the average exchange rates for the 2022 calendar year.^20^

Reporting followed the Consolidated Health Economic Evaluation Reporting Standards 2022 (online supplemental appendix 7).^21^ A detailed reflexivity statement exploring the authorship of this piece is presented in online supplemental appendix 8.

### Data analysis

Data for each facility were presented separately based on primary data collected. Where ranges were reported, a midpoint was selected. Staff salary costs were calculated by multiplying reported full-time salary by the number of full-time equivalent staff. Based on the discussions between the research team and national researchers, the approximate average midwife salary of US$200 per month, identified by the country liaison researchers, was applied to Uganda due to the variability of costs reported by sites. Similarly, for site 3 in Pakistan, an average midwife salary from the other three sites was applied.

Costs of MLBCs were presented as total annual costs for the facility, and these were also divided by the number of births to present a cost per birth for each facility. For the cost analysis, the total health service and total user costs were identified and summed to present a total cost for each model of care. Total DALYs lost were also summed for each model of care. An incremental cost-effectiveness ratio (ICER) was identified by dividing the difference in the total costs of MLBCs and standard care by the difference in DALYs lost from MLBCs and standard care. All results were presented separately for each site and were designed to describe the costs and cost-effectiveness compared with standard care of MLBCs based on that site’s operation. All analyses were conducted using Microsoft Excel.

### Uncertainty analysis

We conducted one-way sensitivity analysis based on cost data reported as zero in the study countries. This included facility, midwife salary, medical officer salary, recruitment and training, and transport costs.

## Results

### Bangladesh

The annual number of births for the four selected MLBC sites in Bangladesh ranged from 101 to 2189 per year. Total annual costs ranged from US$5068 (site 2; 101 births per year) to US$117 662 (site 4; 337 births per year) (online supplemental appendix 9). Total costs per birth were highest at site 4—US$349 per birth; and lowest at site 3—US$21 per birth. Costs were mostly driven by staff salaries and facility operation costs. Facility operation costs per woman were generally higher in smaller facilities as were the midwife salary costs per woman. Site 2, which had 101 births per year, only reported midwife salary costs, with no costs for other staff.

In the modelled cost-effectiveness analysis of MLBCs compared with standard care in Bangladesh, total costs of care (including costs associated with transfers and caesarean births in other facilities) for MLBCs ranged from US$23 439 to US$469 100 (table 1). Costs for standard care were US$314 754 for 1000 women. Sites 1, 2 and 3 had better health outcomes in the total number of DALYs lost than standard care. Sites 1, 2 and 3 produced better outcomes at a lower cost than standard care. Site 4 produced comparable health outcomes to standard care, at higher cost. These additional costs are largely due to the site being extremely remote and it being necessary to pay higher salaries to recruit and retain staff.

**Table 1:** Modelled cost-effectiveness of midwife-led birth centre sites compared with current standard care, Bangladesh, hypothetical cohort of 1000 women

	Standard care	Site 1	Site 2	Site 3	Site 4
**Population size**	1000	1000	1000	1000	1000
Number transferred to other facility	–	600	297	8	208
**Health outcomes**					
Vaginal birth in MLBC	–	400	703	992	792
Vaginal birth in other facility	771	562	119	6	160
Caesarean birth	229	38	178	2	47
No maternal morbidity	967	990	960	986	872
Maternal morbidity	31	10	40	14	128
Maternal death	1	0	0	0	0
Stillbirth/neonatal death	37	0	0	0	12
**DALYS**					
No maternal morbidity	0	0	0	0	0
Maternal morbidity	9	3	11	4	36
Maternal death	1	0	0	0	0
Stillbirth/neonatal death	37	0	0	0	12
Total DALYs lost	47	3	11	4	47
**Provider costs**					
MLBC facility costs	–	US$26 026	US$50 181	US$20 988	US$352 105
Transfer costs	–	US$6843	US$3386	US$86	US$51 636
Other facility costs—vaginal birth	US$62 451	US$45 514	US$9624	US$476	US$12 979
Other facility costs—caesarean birth	US$24 045	US$4029	US$18 713	US$176	US$4985
**Total provider costs**	US$86 496	US$82 413	US$81 903	US$21 726	US$421 705
**User costs**					
MLBC vaginal birth	–	US$0	US$0	US$0	US$0
Other facility vaginal birth	US$140 322	US$102 266	US$21 624	US$1069	US$29 163
Other facility caesarean birth	US$87 936	US$14 736	US$68 436	US$644	US$18 231
**Total user costs**	US$228 258	US$117 001	US$90 059	US$1713	US$47 395
**Total costs (provider and user)**	US$314 754	US$199 414	US$171 963	US$23 439	US$469 100
**ICER**		Dominant (better outcomes, less costly)	Dominant (better outcomes, less costly)	Dominant (better outcomes, less costly)	Same outcomes, more costly

Costs are presented in 2022 US dollars.

DALY, disability-adjusted life-year; ICER, incremental cost-effectiveness ratio; MLBC, midwife-led birth centre.

### Pakistan

The annual number of births for MLBC sites in Pakistan ranged from 95 to 5183 per year. Total annual costs ranged from US$4907 (site 3; 95 births per year) to US$288 649 (site 1; 544 births per year) (online supplemental appendix 10). Total costs per birth were highest at site 1—US$531 per birth; and lowest at site 4—US$34 per birth. Costs were mostly driven facility operation, equipment purchase and other staff costs. Midwife staffing costs ranged from US$6 per woman at site 2 to US$42 per woman at site 1, however, this was less than the amount spent on other staff at sites 1, 2 and 4.

In the modelled cost-effectiveness analysis of MLBCs compared with standard care, total costs of care for MLBCs in Pakistan ranged from US$36 519 to US$693 521 (table 2). Costs for standard care were US$176 057 for 1000 women. Sites 2 and 4 produced better outcomes at lower cost than standard care. Site 3 produced poorer outcomes and was more costly than standard care. Based on costs and outcomes of site 1, MLBCs would cost an additional US$7392 per DALY averted.

**Table 2:** Modelled cost-effectiveness of midwife-led birth centre sites compared with current standard care, Pakistan, hypothetical cohort of 1000 women

	Standard care	Site 1	Site 2	Site 3	Site 4
**Population size**	1000	1000	1000	1000	1000
Number transferred to other facility	–	368	39	368	5
**Health outcomes**					
Vaginal birth in MLBC	–	632	961	632	995
Vaginal birth in other facility	769	184	9	284	1
Caesarean birth	231	184	30	84	4
No maternal morbidity	989	985	993	558	992
Maternal morbidity	10	15	7	442	8
Maternal death	2	0	0	0	0
Stillbirth/neonatal death	70	0	35	32	0
**DALYs**					
No maternal morbidity	0	0	0	0	0
Maternal morbidity	3	4	2	123	2
Maternal death	1	0	0	0	0
Stillbirth/neonatal death	70	0	35	32	0
Total DALYs lost	74	4	37	155	2
**Provider costs**					
MLBC facility costs	–	US$531 506	US$49 452	US$51 648	US$33 813
Transfer costs	–	US$23 779	US$2496	US$23 829	US$352
Other facility costs—vaginal birth	US$30 760	US$14 890	US$735	US$23 021	US$110
Other facility costs—caesarean birth	US$37 422	US$19 301	US$3100	US$8842	US$429
**Total provider costs**	US$68 182	US$589 476	US$55 782	US$107 340	US$34 704
**User costs**					
MLBC vaginal birth	–	–	–	–	–
Other facility vaginal birth	US$60 751	US$33 456	US$1650	US$51 726	US$248
Other facility caesarean birth	US$47.124	US$70 588	US$11 336	US$32 337	US$1567
**Total user costs**	US$107 875	US$104 044	US$12 986	US$84 063	US$1815
**Total costs (provider and user)**	US$176 057	US$693 521	US$68 768	US$191 404	US$36 519
**ICER**	–	US$7392/DALY averted	Dominant (better outcomes, less costly)	Dominated (poorer outcomes, more costly)	Dominant (better outcomes, less costly)

Costs are presented in 2022 US dollars.

DALY, disability-adjusted life-year; ICER, incremental cost-effectiveness ratio; MLBC, midwife-led birth centre.

### Uganda

The annual number of births for MLBC sites in Uganda ranged from 12 to 1242 per year. Total annual costs ranged from US$7922 (site 4; 64 births per year) to US$348 000 (site 3; 1242 births per year) (online supplemental appendix 11). Total costs per birth were highest at site 2—US$2374 per birth, although this site cannot be considered typical. Site 2 is in a remote area and is supported by wealthy donors prepared to pay for equipment and four full-time midwives, even though there were only 12 births in the past twelve months. Total costs per birth were lowest at site 4—US$124 per birth. Costs were mostly driven by facility operations costs, midwife salaries and other staff salaries. Midwife staffing costs ranged from US$10 per woman at site 3 with 1242 births per annum to US$800 per woman at site 2 with just 12 births. Other staff salary costs ranged from US$232 per woman (site 3) to US$23 per woman (site 1). Sites 1 and 3 did not report any equipment costs.

In the modelled cost-effectiveness analysis for Uganda, total costs of care ranged from US$147 273 (site 4) to US$2 458 750 (site 2) (table 3). Costs for standard care were US$277 012 for 1000 women. In terms of cost-effectiveness, sites 1, 2 and 3 MLBCs would lead to better health outcomes than standard care. Site 1 delivered better outcomes at a lower cost than standard care, site 3 had a small ICER of US$571 per DALY saved, and site 2 had a larger ICER of US$55 942 per DALY saved. Site 4 demonstrated lower costs, but poorer health outcomes compared with standard care.

**Table 3:** Modelled cost-effectiveness of midwife-led birth centre sites compared with current standard care, Uganda, hypothetical cohort of 1000 women

	Standard care	Site 1	Site 2	Site 3	Site 4
**Population size**	1000	1000	1000	1000	1000
Number transferred to other facility	–	124	250	19	63
**Health outcomes**					
Vaginal birth in MLBC	–	876	750	981	938
Vaginal birth in other facility	938	106	250	10	31
Caesarean birth	62	18	0	10	31
No maternal morbidity	988	1000	1000	976	984
Maternal morbidity	8	0	0	24	16
Maternal death	3	0	0	0	0
Stillbirth/neonatal death	34	27	0	13	47
**DALYs**					
No maternal morbidity	0	0	0	0	0
Maternal morbidity	2	0	0	7	4
Maternal death	3	0	0	0	0
Stillbirth/neonatal Death	34	27	0	13	47
Total DALYs lost	39	27	0	20	51
**Provider costs**					
MLBC facility costs	0	US$172 673	US$2 384 900	US$280 193	US$123 773
Transfer costs	0	US$0	US$8100	US$403	US$0
Other facility costs—vaginal Birth	US$75 978	US$8602	US$20 250	US$783	US$2531
Other facility costs—caesarean birth	US$6510	US$1858	-	US$1014	US$3281
**Total provider costs**	US$82 488	US$183 133	US$2 413 250	US$282 393	US$129 586
**User costs**					
MLBC vaginal birth	–	–	–	–	–
Otherfacility vaginal birth	US$170 716	US$19 327	US$45 500	US$1758	US$5688
Other facility caesarean birth	US$23 808	US$6796	-	US$3710	US$12 000
**Total user costs**	US$194 524	US$26 124	US$45 500	US$5469	US$17 688
**Total costs (provider and user)**	US$277 012	US$209 257	US$2 458 750	US$287 862	US$147 273
**ICER**	–	Dominant (better outcomes, less costly)	US$55 942/DALY averted	US$571/DALY averted	Poorer health outcomes, less costly

Costs are presented in 2022 US dollars.

DALY, disability-adjusted life-year; ICER, incremental cost-effectiveness ratio; MLBC, midwife-led birth centre.

### Cross-country comparison

**Figure 1:**
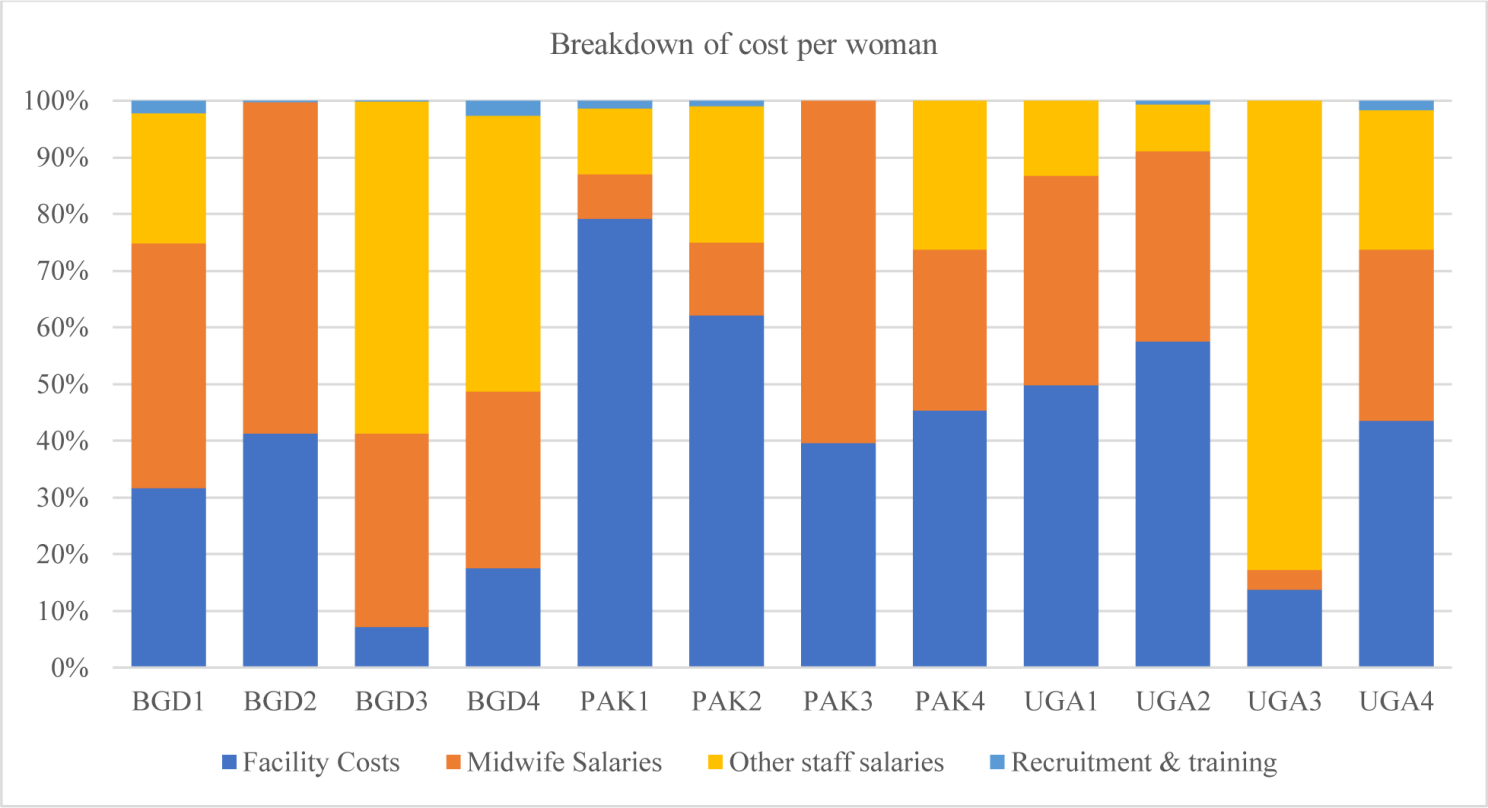
Proportion of total costs attributable to facility costs, midwife salaries, other staff salaries, recruitment and training for midwife-led birth centres in Bangladesh, Pakistan and Uganda in 2022 US dollars. BGD, Bangladesh; PAK, Pakistan; UGA, Uganda.

**Figure 2:**
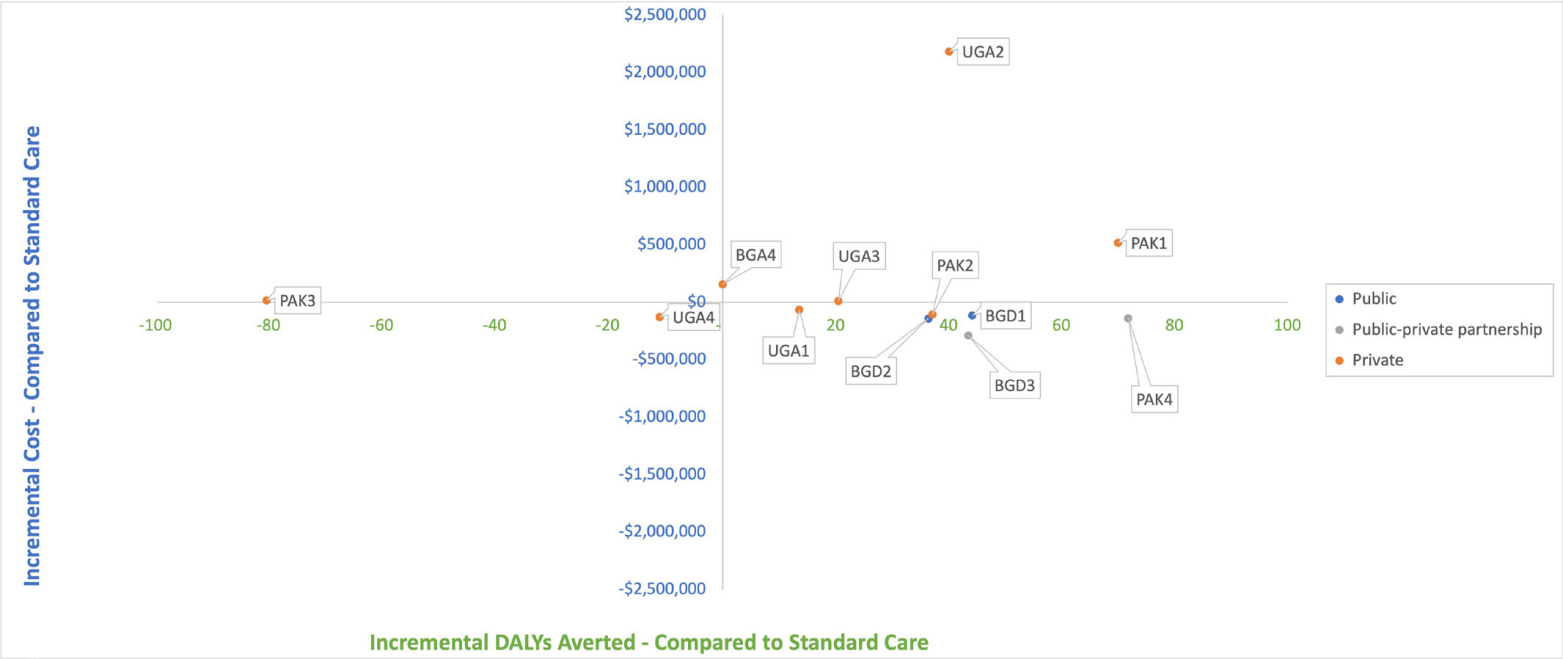
Incremental cost-effectiveness of midwife-led birth centres compared with standard care, Bangladesh, Pakistan, Uganda in 2022 US dollars. BGD, Bangladesh; PAK, Pakistan; UGA, Uganda.

There was no discernible pattern between facility size and cost per birth, with both larger and smaller facilities reporting low costs per birth (online supplemental appendix 12). Midwife salary costs and facility costs were generally the largest contributor to overall costs across each of the sites and countries (figure 1). Sites 3 and 4 in Bangladesh and site 3 in Uganda were notable exceptions to this, with most costs being attributable to other staff salaries. From the modelled cost-effectiveness analysis, all public and public–private partnership MLBCs produced better health outcomes and were less costly than standard care (figure 2). In total, 9 of the 12 (75%) of the sites produced better health outcomes than standard care, as measured by DALYs; and half (6 of the 12 MLBCs) produced better health outcomes and were cost saving.

Results of the sensitivity analysis are presented in online supplemental appendix 13. Replacing data reported as having zero costs with country averages did not substantially change the ICERs produced.

## Discussion

Using a case study approach, this economic evaluation identified the range of reported costs of operating MLBCs in 12 sites in Bangladesh, Pakistan and Uganda, and estimated their cost-effectiveness relative to standard care. Costs of operating MLBCs within the countries varied greatly. Midwife salaries and annual facility operation costs were consistent cost drivers in all countries. In the modelled cost-effectiveness analysis, 6 of the 12 MLBCs were ‘dominant’, producing both better health outcomes and lower costs compared with standard care. Two of the remaining sites had an ICER of less than US$8000 per DALY averted, meaning it would cost less than US$8000 to prevent one additional DALY using MLBCs.

Our study is the first to quantify the costs of MLBCs outside of high-income country settings, making identification of comparable studies difficult due to differences in health systems characteristics. Nonetheless, our findings are consistent with a retrospective cohort study of more than 364 000 births in Australia between 2001 and 2012, which also found MLBCs resulted in lower costs than other models of care.^22^ The Birthplace in England study also found similar results.^23^ Other studies have also found that expanding access to midwife-led care can substantially reduce maternal and neonatal mortality and morbidity rates and improve maternal and newborn health and well-being.^24 25^ Our findings show MLBCs can be cost-saving or cost-effective relative to other models of care, and thus appear to be broadly consistent with results from other settings. We did note that costs of operation and cost-effectiveness varied widely between and within countries, and cost-effectiveness does appear to be dependent on the unique local site characteristics as opposed to general characteristics such as size or rurality. Our methodology also highlighted the importance of close collaboration with local health service team to identify the context of expenditure. We identified MLBCs that demonstrated better health outcomes and cost savings in all three countries, private, public and public–private partnerships, rural and urban settings, and in freestanding as well as in those onsite with or alongside referral facilities.

MLBCs can help meet the growing demand for facility-based birth for low-risk women and might be particularly beneficial in LMICs where universal access to higher level facility-based care is limited.^26^ Shifting the main strategy for reducing maternal and neonatal mortality in many LMICs from increasing the rate of deliveries within medical facilities^4^ to focusing on the quality of healthcare may better translate to better maternal and neonatal health outcomes.^5 24^ Clinical findings showing that care provided in MLBCs is as safe and effective as that in the obstetric units and results in less intervention justify the expansion of this model of care so that scarce resources can be used more effectively.^27^ This study provides evidence for MLBCs in LMICs as an effective, evidence-based strategy to improve the quality, costs and experiences of maternity care. Further, there did not seem to be a clear scale efficiency effect, indicating that MLBCs could be cost-effective at small or larger scales in LMIC settings, once contextual factors are considered.

### Strengths and limitations

A key strength of this study was that data were collected from a range of sites and countries in a real-world setting to identify variation in costs and outcomes. We codesigned our data collection tool with country researchers to comprehensively capture the range of operation costs. Nonetheless, our study was limited by the inability of some sites to identify some areas of expenditure—particularly equipment costs and midwife salaries. Furthermore, as all facilities were already established, we were unable to identify set-up costs. A key recommendation from this study is investment in prospective implementation analysis of the costs and outcomes produced when new MLBCs are established in LMICs, as well as investment in standardised data capture tools for identifying costs and outcomes. As such, the results of this study must be interpreted with caution as they are reliant on the accuracy of the reported data in a small number of sites.

Our analysis was also unable to capture some additional benefits of MLBCs beyond mortality and morbidity, particularly around women’s experiences and satisfaction with care—which are key to capturing the full value associated with midwife-led care.^28^ A key component for all MLBCs and midwife-led care more broadly, is the woman-centred philosophy, continuity of care during pregnancy and after birth, and involvement of women in all decisions regarding perinatal care.^14 22 29–31^ MLBCs seek to promote normal, physiological childbirth by recognising, respecting and safeguarding normal birth processes through individualised care,^32^ as opposed to the typical hospital approach to labour which is much more time-oriented and standardised, and not infrequently, there is a pressure on midwives to accelerate the process by carrying out unnecessary medical intervention.^33^ Consequently, women who give birth in an MLBC report feeling supported in their ability to participate in the decision-making process, greater autonomy, and, thus, greater acceptance of and satisfaction with perinatal care in this setting among pregnant women.^22 30–32 34 35^ These additional benefits were unable to be captured in our study but are important to recognise when considering the value of MLBCs.

## Conclusion

MLBCs offer a potentially cost-effective model of care for providing safe and high-quality care to women giving birth in LMICs. However, the cost of operating an MLBC varies greatly, and this does affect cost-effectiveness. Further research, including prospective evaluation of implementation of new MLBCs, is recommended to confirm the results produced in our study.

## Data Availability

No data are available. Ethics approval prohibits data sharing.
