# Supplementary material for: Midwife-led birthing centres in Bangladesh, Pakistan and Uganda: an economic evaluation of case study sites
Source: BMJ Glob Health. 2024 Mar 28;9(3):e013643. doi: 10.1136/bmjgh-2023-013643 (PMC10982789; doi:10.1136/bmjgh-2023-013643)
Supplement: Supplementary data [file bmjgh-2023-013643supp001.pdf]

Table of contents

Appendix 1: Case study site characteristics	Page 1
Appendix 2: Co-designed cost data collection tool	Page 1
Appendix 3: Decision tree structure	Page 3
Appendix 4: Health outcomes produced with current 'standard care' in each country	Page 3
Appendix 5: Costs by mode of birth in non-midwife-led birth centre facilities in each country, 2022 United States Dollar	Page 4
Appendix 6: Disability weights, and disability-adjusted life year or years of life lost for maternal morbidity, maternal mortality, stillbirth, and neonatal mortality over a one-year time horizon	Page 4
Appendix 7: CHEERS 2022 Checklist	Page 4
Appendix 8: Structured reflexivity statement	Page 6
Appendix 9: Annual operation costs of midwife-lid birth centre facilities in Bangladesh, 2022 United States Dollars	Page 8
Appendix 10: Annual operation costs of midwife-led birth centres facilities in Pakistan, 2022 United States Dollars	Page 9
Appendix 11: Annual operation costs of midwife-led birth centre facilities in Uganda, 2022 United States Dollars	Page 9
Appendix 12: Annual number of births, and costs per birth (in 2022 United States Dollars) for midwife-led birth centres in Bangladesh, Pakistan, and Uganda	Page 9-10
Appendix 13: One-way sensitivity analysis	Page 11

Appendix 1: Case study site characteristics

Country	Site number	Location	Rural/ Urban location	Distance from referral facility (km)	Sector	Type
Bangladesh	1	Rajshahi	Rural	0	Public	Onsite/ alongside
	2	Sylhet	Rural	19	Public	Freestanding
	3	Sylhet	Rural	1	Public-private partnership	Freestanding
	4	Chittagong	Rural	34	Private	Freestanding
Pakistan	1	Punjab	Rural	No formal referral arrangements	Private	Freestanding
	2	Sindh	Urban	0	Private	Onsite/ alongside
	3	Balochistan	Rural	No formal referral arrangements	Private	Freestanding
	4	Sindh	Rural	No formal referral arrangements	Public-private partnership	Freestanding
Uganda	1	Central	Rural	7	Private	Freestanding
	2	Eastern	Rural	45	Private	Freestanding
	3	Northern	Rural	70	Private	Freestanding
	4	Western	Rural	10	Private	Freestanding

Notes: 'Private sector' includes for-profit, not-for-profit, and non-governmental organisation. 'Freestanding' means on a site separate from a hospital or other type of health facility to which complicated cases may be referred. Several of the freestanding MLBCs were on the same site as another health facility (e.g., a primary health centre), but not on the same site as a referral facility. 'Onsite/alongside' means on the same site as a hospital or other type of health facility to which complicated cases may be referred. The urban/rural classification was as defined by the national research teams.

Appendix 2: Co-designed cost data collection tool

**Name of country**	
**Name of site**	
**Timeframe of data collection**	
**Number of women booked at the MLBC per year**	

Callander EJ, *et al. BMJ Glob Health* 2024; 9:e013643. doi: 10.1136/bmjgh-2023-013643

**Antenatal period**	Example
Do you provide antenatal care?	Yes/no
Number of women booked at the MLBC per year (eg. 12 months: January 2021 to January 2022)	
Average number of antenatal visits provided per woman	
**Intrapartum period**	
Number of births in the MLBC (actual births in the MLBC)	
Number of women transferred to a higher facility (Transfers for higher level care in total)	
Transferred during labour (number of women transferred in labour)	
Transferred after the birth (number women transferred after the birth)	
Number of babies transferred to a higher facility	
Number of women with assisted vaginal birth	
Number of women with caesarean birth after transfer from MLBC	
Number of women with 3rd/4th degree perineal trauma	
Number of women with postpartum haemorrhage (>600mL)	
Number of women with other significant complications (please specify the complications eg. Obstructed labour, sepsis, obstetric fuistula, eclampsia)	
Complication	
Complication	
Complication (add rows as needed)	
Number of maternal deaths (In the MLBC)	
Number of stillbirths (In the MLBC)	
Number of early neonatal deaths <7 days	
Number of late neonatal deaths <28 days	
Number of postnatal visits in the community (average number of visits per woman multiplied by the number of women)	
Average estimated length of stay (Average length of stay in the MLBC)	
**Cost estimates**	
Cost of new staff recruitment per year (Costs associated with staff recruitment if any)	
Midwifery staff	
Administrative staff	
Medical staff	
Other staff (add rows as needed)	
Cost of staff training - new (Costs associated with staff training if any: emergency procedures, additional skills)	
Cost of staff training - ongoing (Costs associated with staff training if any: emergency procedures, additional skills)	
Annual equipment purchase budget (eg. Birth packs, beds, furniture)	
Annual equipment hire budget	
Annual facility hire and/or purchase costs (Hire or purchase of buildings)	
Monthly facility operation costs (Includes electricity, water etc)	
**Staffing**	
Average number of midwives working at MLBC per month (FTE) (Full time equivalent)	
Monthly salary of midwifery staff	
Average number of other staff working at the MLBC over a typical month (FTE) (Administration staff, maintenance staff, managerial staff, medical staff. Please specify)	
Other staff type 1	
Other staff type 2	
Other staff type 3 (add rows if needed)	

Monthly salary of other staff (Full time monthly salary of other staff, please specify)	
Other staff type 1	
Other staff type 2	
Other staff type 3 (add rows if needed)	
**Other costs**	
Cost of transport for women to higher level facility (cost)	
Mode of transport for transfer (eg ambulance, private care)	

Appendix 3: Decision-tree structure

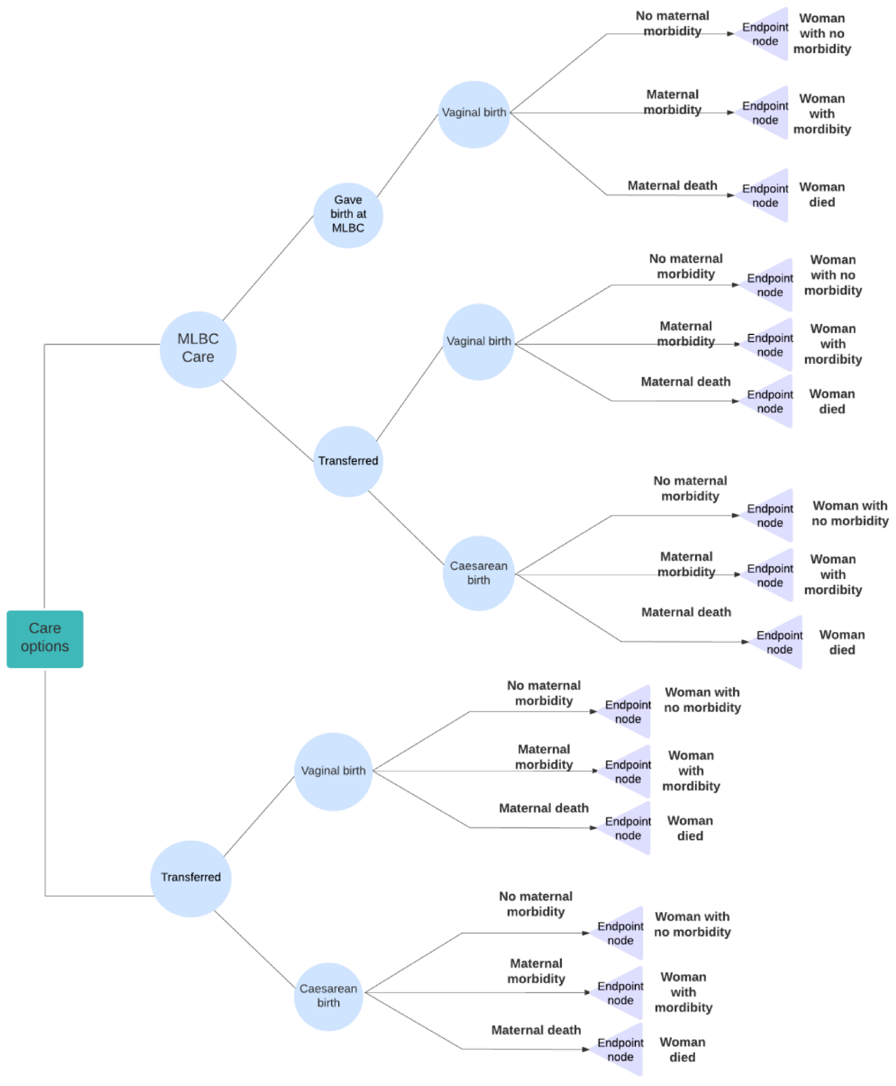

Appendix 4: Health outcomes produced with current 'standard care' in each country

	Caesarean Birth (% of births)*	Maternal Morbidity^	Maternal Mortality#	Stillbirth*	Neonatal Death*
**Bangladesh**	36%	31.33 per 1000	123 per 100000	21 per 1000	16 per 1000
**Pakistan**	23.1%	9.52 per 1000	154 per 100000	31 per 1000	39 per 1000
**Uganda**	6.2%	8.42 per 1000	284 per 100000	15 per 1000	19 per 1000

*Source: (1)

#Source: (2)

^Source: (3, 4)

Appendix 5: Costs by mode of birth in non-midwife-led birth centre facilities in each country, 2022 United States Dollar

	Caesarean birth - hospital	Caesarean birth – user charge	Vaginal birth – hospital	Vaginal birth – user charge
**Bangladesh***	$105	$384	$81	$182
**Pakistan^**	$162	$204	$40	$79
**Uganda#**	$123	$56	$33	$26

*Source: Kawnine, Guinness (5). Reported cost of caesarean birth in Bangladesh for three facilities, and the mean cost was used for the analysis. For vaginal birth, the same cost with the cost of operation charge, anaesthetic, anaesthetist, and surgeon was used.

^Source: (6)

#Source: (7)

Appendix 6: Disability weights, and disability-adjusted life year or years of life lost for maternal morbidity, maternal mortality, stillbirth, and neonatal mortality over a one-year time horizon

	Disability Weight*	Length of time (years)	DALY/YLL
**No maternal morbidity**	0	1	0
**Maternal morbidity**	0.2787	1	0.2787
**Maternal death**	1	1	1
**Stillbirth**	1	1	1
**Neonatal death**	1	1	1

*Source: (8)
Note: Disability adjusted life year (DALY); Years of life lost (YLL)

Appendix 7: CHEERS 2022 Checklist

Topic	No.	Item	Location where item is reported
**Title**			
	1	Identify the study as an economic evaluation and specify the interventions being compared.	Pg 1
**Abstract**			
	2	Provide a structured summary that highlights context, key methods, results, and alternative analyses.	Pg 2
**Introduction**			
**Background and objectives**	3	Give the context for the study, the study question, and its practical relevance for decision making in policy or practice.	Pg 3
**Methods**			

Topic	No.	Item	Location where item is reported
**Health economic analysis plan**	4	Indicate whether a health economic analysis plan was developed and where available.	Pg 3
**Study population**	5	Describe characteristics of the study population (such as age range, demographics, socioeconomic, or clinical characteristics).	Pg 3, Appendix 1
**Setting and location**	6	Provide relevant contextual information that may influence findings.	Pg 3
**Comparators**	7	Describe the interventions or strategies being compared and why chosen.	Pg 3
**Perspective**	8	State the perspective(s) adopted by the study and why chosen.	Pg 4
**Time horizon**	9	State the time horizon for the study and why appropriate.	Pg 4
**Discount rate**	10	Report the discount rate(s) and reason chosen.	Pg 4
**Selection of outcomes**	11	Describe what outcomes were used as the measure(s) of benefit(s) and harm(s).	Pg 3-4
**Measurement of outcomes**	12	Describe how outcomes used to capture benefit(s) and harm(s) were measured.	Pg 3-4, Appendix 4
**Valuation of outcomes**	13	Describe the population and methods used to measure and value outcomes.	Pg 3-4, Appendix 6
**Measurement and valuation of resources and costs**	14	Describe how costs were valued.	Pg 4, Appendix 5
**Currency, price date, and conversion**	15	Report the dates of the estimated resource quantities and unit costs, plus the currency and year of conversion.	Pg 4
**Rationale and description of model**	16	If modelling is used, describe in detail and why used. Report if the model is publicly available and where it can be accessed.	Pg 4, Appendix 3
**Analytics and assumptions**	17	Describe any methods for analysing or statistically transforming data, any extrapolation methods, and approaches for validating any model used.	N/a
**Characterising heterogeneity**	18	Describe any methods used for estimating how the results of the study vary for subgroups.	N/a
**Characterising distributional effects**	19	Describe how impacts are distributed across different individuals or adjustments made to reflect priority populations.	N/a
**Characterising uncertainty**	20	Describe methods to characterise any sources of uncertainty in the analysis.	Pg 5

Topic	No.	Item	Location where item is reported
**Approach to engagement with patients and others affected by the study**	21	Describe any approaches to engage patients or service recipients, the general public, communities, or stakeholders (such as clinicians or payers) in the design of the study.	Pg 4
**Results**			
**Study parameters**	22	Report all analytic inputs (such as values, ranges, references) including uncertainty or distributional assumptions.	Appendix 4-6
**Summary of main results**	23	Report the mean values for the main categories of costs and outcomes of interest and summarise them in the most appropriate overall measure.	Appendix 9-11
**Effect of uncertainty**	24	Describe how uncertainty about analytic judgments, inputs, or projections affect findings. Report the effect of choice of discount rate and time horizon, if applicable.	Pg 6, Appendix 12
**Effect of engagement with patients and others affected by the study**	25	Report on any difference patient/service recipient, general public, community, or stakeholder involvement made to the approach or findings of the study	Pg 7
**Discussion**			
**Study findings, limitations, generalisability, and current knowledge**	26	Report key findings, limitations, ethical or equity considerations not captured, and how these could affect patients, policy, or practice.	Pg 6-7
**Other relevant information**			
**Source of funding**	27	Describe how the study was funded and any role of the funder in the identification, design, conduct, and reporting of the analysis	Pg 7
**Conflicts of interest**	28	Report authors conflicts of interest according to journal or International Committee of Medical Journal Editors requirements.	Pg 7

Note: Source (9)

Appendix 8: Structured reflexivity statement

1. How does this study address local research and policy priorities?

This study was commissioned by the Bill and Melinda Gates Foundation and led by the International Confederation of Midwives (ICM). ICM has over 139 members, representing midwifery associations in around 118 countries across the 6 regions of the world. The study was undertaken by Novametrics and The Burnet Institute, based in the UK and Australia respectively with partners in Bangladesh, South Africa, Pakistan, and Uganda. The objective of the wider study was to provide examples of well-functioning midwife-led birth centres (MLBC) in low- to middle-income countries with the view to developing a strategy to upscale these services for women in settings where sexual and reproductive health care services are limited. This aspect of the study provides an economic evaluation of MLBCs to inform the financial feasibility of establishing these services.

2. How were local researchers involved in study design?

Research teams from the three countries included in this paper were instrumental in the design and execution of the data collection and drafting of the manuscript. Researchers from Bangladesh (AA, AH, FR) Uganda (SM, RN), and Pakistan (IR, AT) led the collaboration with Novametrics and the Burnet Institute in the development of the data collection tools specifically designed for this study. These in-country researchers undertook all the data collection and assisted with data verification and analysis. The wider research team also included researchers from Rwanda (OB) and South Africa (SC) along with members from the UK (AN, MB), Australia (CH, EC, VS, ST), and the ICM (MF, SM, SP).

3. How has funding been used to support the local research team?

Each of the research teams in the four countries received funding from the grant held through ICM to employ their teams. There was a research leader in each country, and they identified early and mid-career researchers who were then paid through the grant. Each country provided a budget to suit their requirements and received funding. Subsequent to this paper, the in-country teams have received additional funding to further develop country-specific papers which they have led.

4. How are research staff who conducted data collection acknowledged?

All members of the research teams are co-authors of this paper.

5. Do all members of the research partnership have access to study data?

All members of the partnership had access to data.

6. How was data used to develop analytical skills within the partnership?

Given the data analysis involved the synthesis of all the included countries, the main analysis was conducted in Australia by a health economist. However, all in-country team members were consulted on the validity and accuracy of the data collected and the interpretation of their data. A number of meetings were held with the teams during the analysis and writing of this paper. Several of the in-country researchers requested additional information about the analysis methods used for this paper, and one-to-one meetings were held with them to provide this additional information. International team members have published or are also planning to publish papers based on the data collected in their own countries during this study.

7. How have research partners collaborated in interpreting study data?

Several meetings were held with the individual international teams to discuss the raw data and the outcomes found. These team members were also involved in writing the manuscript.

8. How were research partners supported to develop writing skills?

The research partners were supported especially by the team leaders (AN and CH) to refine their writing skills. This was done over several stages. First, the teams were asked to prepare an abstract for a paper based on their own country's data. CH and AN provided feedback on the abstract, after which each in-country team prepared a first draft of their manuscript. Between 3-4 rounds of feedback were provided for each country team before the manuscripts were submitted to a journal.

9. How will research products be shared to address local needs?

The results of this and other aspects of the wider research project will inform projects to expand the availability of midwife-led birth centres in low- and middle-income countries. We have already presented the overall project at two international conferences, and research partners in the 4 countries each presented different aspects of the overall project. We have also conducted a series of webinars for ICM member associations and midwives from around the world. The in-country partners led different parts of these webinars. The webinars attracted over 2000 registrants, mostly from LMICs.

10. How is the leadership, contribution and ownership of this work by LMIC researchers recognised within the authorship?

Authors EC and MB worked as part of the senior authorship team in developing this manuscript, and their contribution has been recognised as joint first and joint last authors respectively. All in-country researchers

involved in the economic analysis have been included in the authorship. Nine of the 19 authors are based in high-income countries. However, this paper is one of several products from the larger study and the international team members have published or are also planning to publish papers based on the data collected in their own countries during this study.

11. How have early career researchers across the partnership been included within the authorship team?

There are two ECRs (VS, ST) based in Australia involved in this project and are authors on this paper. There were also ECRs from the international team included.

12. How has gender balance been addressed within the authorship?

Thirteen of the authors are female (EC, VS, AN, CH, AC, SC, SM, RN, OB, ST, MF, SM, SP) and the remaining six are male (AA, AH, FR, SR, AT, MB).

13. How has the project contributed to training of LMIC researchers?

The researchers from the LMICs received ongoing support and encouragement in the design, data collection, analysis and to support the writing. In particular, the in-country teams have been supported by the researchers from Novametrics and Burnet to develop their capacity in writing for publication in academic journals, preparing for conference presentations and conducting economic analyses. Many have commented that the experience was very positive, and they learned a lot.

14. How has the project contributed to improvements in local infrastructure?

This project has not directly contributed to improvements in local infrastructure. It is, however, hoped that the results will be used in the future to advocate for further investment in midwife-led birthing centres in the focus countries and elsewhere.

15. What safeguarding procedures were used to protect local study participants and researchers?

The data collected in-country consisted of service-based routinely collected data and financial service data. There were no local study participants per se, however it was necessary for the researchers to liaise with service providers to obtain the data. The project was covered by human research ethical committee (HREC) approval in each participating country and an overall approval from an approved HREC in Australia.

Appendix 9: Annual operation costs of midwife-lid birth centre facilities in Bangladesh, 2022 United States Dollars

	**Site 1**		**Site 2**		**Site 3**		**Site 4**	
	Total	*Per Woman*	*Total*	*Per Woman*	*Total*	*Per Woman*	*Total*	*Per Woman*
**Annual Number of Births**	2,189		101		1,192		337	
% of women transferred	60%		30%		1%		21%	
Facility Costs								
Facility operation	$9,690	$4	$570	$6	$456	$<1	$4,560	$14
Equipment purchase	$2,138	$1	$1,520	$15	$1,330	$1	$8,075	$24
Equipment hire budget	$6,175	$3	$0	$0	0	$0	$8,200	$24
Staff Costs								
Midwife Salaries	$24,624	$11	$2,964	$29	$8,550	$7	$36,951	$110
Other staff salaries	$13,110	$6	$0	$0	$14,649	$12	$57,738	$171
Training new staff	$618	$<1	$0	$0	$0	$0	$1,140	$3
Ongoing staff training	$618	$<1	$14	$<1	$33	$<1	$998	$3
**Total Costs**	$56,972	$26	$5,068	$50	$25,018	$21	$117,662	$349
Other Costs								
User birth charges	$0	$0	$0	$0	$0	$0	$0	$0
Emergency Transport to other facility if needed - paid by facility	-	$11	-	$11	-	$11	-	$11
Emergency Transport to other facility if needed - paid by women	-	-	-	-	-	-	-	-

Callander EJ, *et al. BMJ Glob Health* 2024; 9:e013643. doi: 10.1136/bmjgh-2023-013643

Appendix 10: Annual operation costs of midwife-led birth centres facilities in Pakistan, 2022 United States Dollars

	Site 1		Site 2		Site 3		Site 4	
	Total	*Per Woman*	*Total*	*Per Woman*	*Total*	*Per Woman*	*Total*	*Per Woman*
**Annual Number of Births**	544		5,183		95		735	
% of women transferred	37%		4%		37%		1%	
Facility Costs								
Facility operation	$111,720	$205	$144,413	$28	$1,646	$17	$1,470	$2
Equipment purchase	$101,430	$186	$9,800	$2	$294	$3	$9,800	$13
Equipment hire budget	$15,190	$28	$4,900	$1	$0	$0	$0	$0
Staff Costs								
Midwife Salaries	$22,579	$42	$33,124	$6	$2,966^	$31^	$7,056	$10
Other staff salaries	$33,810	$62	$61,622	$12	$0	$0	$6,527	$9
Training new staff	$2,450	$5	$0	$0	$0	$0	$0	$0
Ongoing staff training	$1,470	$3	$2,450	<$1	$0	$0	$0	$0
**Total Costs**	$288,649	$531	$256,309	$49	$4,907	$52	$24,853	$34
Other Costs								
User birth charges	$0	$0	$0	$0	$0	$0	$0	$0
Emergency Transport to other facility if needed - paid by facility	-	$65	-	$65	-	$65	-	$65

^outlier annual salary due to unique health service characteristics (midwife shortages and need to pay high salary to attract staff), analysis applied average annual midwife salary from other sites

Appendix 11: Annual operation costs of midwife-led birth centre facilities in Uganda, 2022 United States Dollars

	Site 1		Site 2		Site 3		Site 4	
	Total	*Per Woman*	*Total*	*Per Woman*	*Total*	*Per Woman*	*Total*	*Per Woman*
**Annual Number of Births**	113		12		1,242		64	
% of women transferred	12%		25%		2%		6%	
Facility Costs								
Facility operation	$9,720	$86	$9,720	$810	$48,000	$39	$3,240	$51
Equipment purchase	$0	-	$6,750	$563	$0	-	$162	$3
Equipment hire budget	$0	-	$0	-	$0	-	$41	$1
Staff Costs								
Midwife Salaries	$7,200^	$64^	$9,600^	$800^	$12,000^	$10^	$2,400^	$38^
Other staff salaries	$2,592	$23	$2,333	$194	$288,000	$232	$1,944	$30
Training new staff	$0	$0	$81	$7	$0	$0	$0	$0
Ongoing staff training	$0	$0	$0	$0	$0	$0	$135	$2
**Total Costs**	$19,9512	$173	$28,484	$2,374	$348,000	$280	$7,922	$124
Other Costs								
User birth charges	$0	$0	$0	$0	$0	$0	$0	$0
Emergency Transport to other facility if needed - paid by facility	$0	$0	$0	$0	$0	$0	$0	$0
Emergency Transport to other facility if needed - paid by women	-	$0	-	$32	-	$21	-	$0

^due to the inability to identify the annual salary paid by sites, the analysis applied average annual midwife salary identified by country research liaison.

Appendix 12: Annual number of births, and costs per birth (in 2022 United States Dollars) for midwife-led birth centres in Bangladesh, Pakistan, and Uganda

Callander EJ, *et al. BMJ Glob Health* 2024; 9:e013643. doi: 10.1136/bmjgh-2023-013643

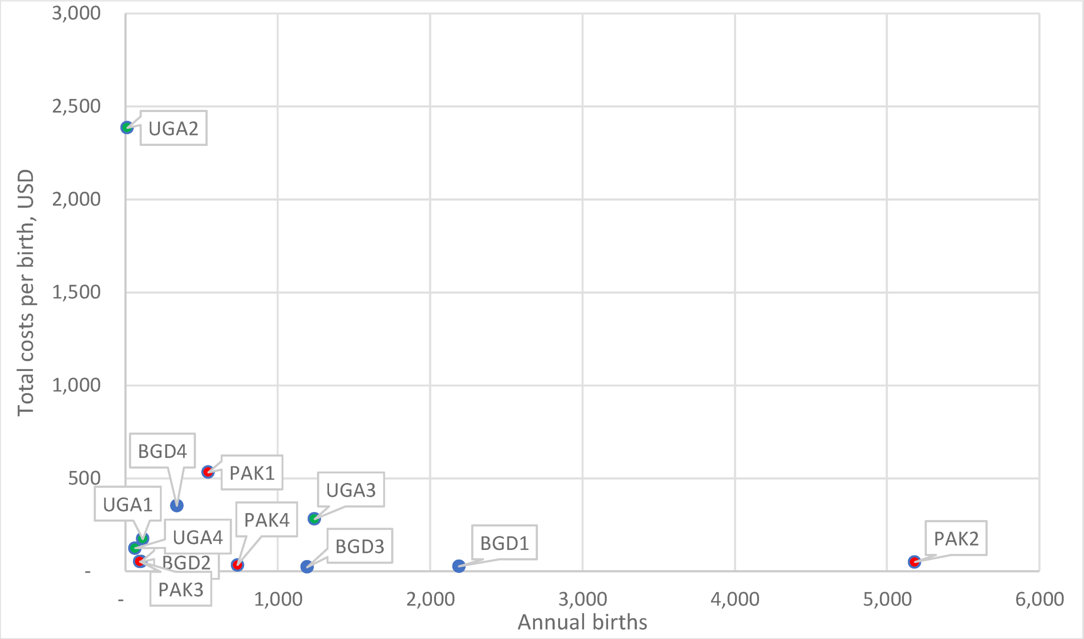

BGD=Bangladesh; PAK=Pakistan; UGA=Uganda

Appendix 13: One-way sensitivity analysis

**ICER sensitivity analysis**	**BGD1**	**BGD2**	**BGD3**	**BGD4**	**PAK1**	**PAK2**	**PAK3**	**PAK4**	**UGA1**	**UGA2**	**UGA3**	**UGA4**
Base case	Dominant (Better outcomes, less costly)	Dominant (Better outcomes, less costly)	Dominant (Better outcomes, less costly)	Same outcomes, more costly	$7,288 /DALY averted	Dominant (Better outcomes, less costly)	Dominated (Poorer outcomes, more costly)	Dominant (Better outcomes, less costly)	Dominant (Better outcomes, less costly)	$55,942 /DALY averted	$571/DALY averted	Poorer health outcomes, less costly
Facility costs: replace 0 with mean country value	No change	No change	No change	No change		No change	No change	No change	$15,899	$54,428 /DALY averted	$14,354 /DALY averted	No change
Midwife salary costs: replace 0 with mean country costs per woman	No change	No change	No change	No change	$7,121 /DALY averted	No change	No change	No change	$7,104	$40,139 /DALY averted	$11,176 /DALY averted	No change
Medical Officer salary costs: use country mean for all MO salaries	No change	No change	No change	No change	$7,088 /DALY averted	No change	No change	No change	No change	No change	No change	No change
Recruitment & training costs: replace 0 with mean country value	No change	No change	No change	No change	No change	No change	No change	No change	No change	$54,465 /DALY averted	$1,512 /DALY averted	No change
Transport costs: replace 0 with mean country value	No change	No change	No change	No change	No change	No change	No change	No change	No change	$54,376 /DALY averted	$535 /DALY averted	No change

References

1. UNICEF. UNICEF Data Warehouse [dataset]. 2022. https://data.unicef.org/resources/data_explorer/unicef_f/.
2. World Health Organization. Trends in maternal mortality 2000 to 2020. Geneva: World Bank 2023.
3. De Silva M, Panisi L, Lindquist A, Cluver C, Middleton A, Koete B, et al. Severe maternal morbidity in the Asia Pacific: a systematic review and meta-analysis. Lancet Reg Health West Pac 2021;14:100217.
4. Nakimuli A, Nakubulwa S, Kakaire O, Osinde MO, Mbalinda SN, Nabirye RC, et al. Maternal near misses from two referral hospitals in Uganda: a prospective cohort study on incidence, determinants and prognostic factors. BMC Pregnancy Childbirth 2016;16:24.
5. Kawnine N, Guinness L, Amin MA, Anwar R, Killingsworth JR, Hedrick-Wong Y, et al. Costs and Outcomes of Caesarean Section Procedures in Public, Private and NGO Health Care Facilities in Bangladesh [online]. 1998. http://oldweb.heu.gov.bd/pdf/Research%20Paper%20No.08.pdf (accessed 17 February 2023).
6. Khan A, Zaman S. Costs of vaginal delivery and Caesarean section at a tertiary level public hospital in Islamabad, Pakistan. BMC Pregnancy Childbirth 2010;10:2.
7. Levin A, Dmytraczenko T, McEuen M, Ssengooba F, Mangani R, Van Dyck G. Costs of maternal health care services in three anglophone African countries. Int J Health Plann Manage 2003;18:3-22.
8. Center for the Evaluation of Value and Risk in Health. DALY Calculator [online]. n.d. https://cevr.shinyapps.io/DALYcalculation/ (accessed 17 February 2023).
9. Husereau D, Drummond M, Augustovski F, et al. Consolidated Health Economic Evaluation Reporting Standards 2022 (CHEERS 2022) Explanation and Elaboration: A Report of the ISPOR CHEERS II Good Practices Task Force. Value Health 2022;25.

Callander EJ, *et al. BMJ Glob Health* 2024; 9:e013643. doi: 10.1136/bmjgh-2023-013643
